# Loss of *popdc3* Impairs Mitochondrial Function and Causes Skeletal Muscle Atrophy and Reduced Swimming Ability in Zebrafish

**DOI:** 10.1002/jcsm.13794

**Published:** 2025-04-16

**Authors:** Chen‐Chen Sun, Zhang‐Lin Chen, Dong Yang, Jiang‐Ling Xiao, Xiang‐Tao Chen, Xi‐Yang Peng, Xiu‐Shan Wu, Chang‐Fa Tang

**Affiliations:** ^1^ Key Laboratory of Physical Fitness and Exercise Rehabilitation, College of Physical Education Hunan Normal University Changsha Hunan China; ^2^ Institute of Physical Education Hunan First Normal University Changsha Hunan China; ^3^ Center for Heart Development, College of Life Sciences Hunan Normal University Changsha China

**Keywords:** mitochondrial dysfunction, POPDC3, protein homeostasis, skeletal muscle atrophy, skeletal muscle mass

## Abstract

**Background:**

The Popeye domain containing 3 (POPDC3) protein is essential for the maintenance of skeletal muscle homeostasis. POPDC3 is a pathogenic variant gene of limb‐girdle muscular dystrophy (LGMD), and its variants lead to LGMDR26. At the animal level, zebrafish larvae with *popdc3* mutations develop tail curls and muscle atrophy. However, the mechanism of skeletal muscle atrophy induced by *POPDC3* variants/loss remains unclear.

**Methods:**

Eight‐month‐old male WT and *popdc3* mKO zebrafish were used for this research. Loli Track (Denwmark) and Loligo Swimming Respirometer were used to observe the zebrafish's swimming ability. The zebrafish skeletal muscle structure and cross‐sectional area (CSA) were observed and counted by transmission electron microscopy (TEM), H&E and wheat germ agglutinin (WGA). Enriched genes and signalling pathways were analysed using RNA sequencing, and the effects of *popdc3* mKO on zebrafish skeletal muscle mitochondrial respiration, biogenesis and dynamics were examined to investigate possible mechanisms.

**Results:**

The swimming ability of *popdc3* mKO zebrafish was reduced, and as evidenced by the reluctance to move, fewer movement trajectories, the total distance travelled (*p* < 0.001), the average velocity of movement (*p* < 0.001), oxygen consumption (MO_2_) (*p* < 0.01), maximum oxygen consumption (MO_2max_) (*p* < 0.05), critical swimming speed (U_crit_) (*p* < 0.01) and relative swimming speed (U_crit‐r_) (*p* < 0.01) were significantly decreased and increased of the exhaustive swimming time (*p* < 0.01). In addition, loss of *popdc3* reduced zebrafish skeletal muscle weight (*p* < 0.001), muscle/body weight (*p* < 0.01), myofibre size and CSA (*p* < 0.01), increased protein degradation (ubiquitination and autophagy) (*p* < 0.05) and decreased protein synthesis (*p* < 0.05), suggesting that *popdc3* deficiency induces zebrafish skeletal muscle atrophy. Further, *popdc3* mKO zebrafish mitochondrial function is reduced, as evidenced by impaired mitochondrial respiration, decreased biogenesis and kinetic imbalance (*p* < 0.05).

**Conclusions:**

POPDC3, a Popeye protein, plays an important role in controlling mitochondrial function and skeletal muscle mass and strength. Loss of *popdc3* decreases mitochondrial respiration and mitochondrial biogenesis, disrupting kinetic homeostasis, which induces mitochondrial dysfunction and impaired protein turnover (reduced synthesis and increased degradation), leading to zebrafish skeletal muscle atrophy.

## 1 Introduction

Skeletal muscle atrophy is characterized by the decline of muscle mass, strength and function, which seriously affects the patient's life quality [1]. According to different aetiology, skeletal muscle atrophy is classified into primary skeletal muscle atrophy (congenital or hereditary) and secondary skeletal muscle atrophy (pathological or physiological) [1]. This is caused by an imbalance between skeletal muscle protein synthesis and degradation, which triggers muscle wasting and leads to atrophy [2]. Limb‐girdle muscular dystrophies (LGMDs) belong to primary skeletal muscle atrophy, a genetically heterogeneous, autosomal inherited muscular dystrophy caused by different gene defects [3]. The direct pathogenic factor is the variants of many genes encoding protein, which results in loss of function or abnormalities of the proteins and then affects the maintenance, repair and normal function of muscle through the influence of extracellular matrix (ECM) and molecular transport, signal transduction pathway and nuclear function [4].

The Popeye domain containing (POPDC) proteins is a family of LGMD pathogenic variants proteins, including three isoforms: POPDC1, POPDC2 and POPDC3, which are expressed at high levels in heart and skeletal muscle [5, 6]. Among them, POPDC1 is expressed at almost equal levels in both types of striated muscle, POPDC2 is strongly expressed in the heart and weakly expressed in skeletal muscle and POPDC3 is predominantly expressed in skeletal muscle [7, 8]. Studies have shown that *POPDC1* variants cause muscular dystrophy and arrhythmia by affecting protein trafficking [9]. Knockdown of *popdc2* affects the development of zebrafish skeletal muscle and heart, resulting in aberrant craniofacial and trunk muscles [10]. Mice deficient in either *Popdc1* or *Popdc2* developed severe stress‐induced cardiac bradycardia [11]. This suggests that the POPDC family plays an important function in the control of striated muscle homeostasis.

In 2019, Vissing et al. [12] reported the function of POPDC3 in skeletal muscle for the first time. It was found that muscle biopsies from five patients carrying different *POPDC3* variants showed features typical of muscular dystrophy. In addition, the 3dpf zebrafish of *popdc3* knockdown were found with features of tail curling and dystrophic muscle. These findings suggest that the POPDC3 was associated with a new LGMD. Subsequently, Ullah et al. [5] revealed an association between *POPDC3* variants and LGMD, whole‐exome sequencing (WES) analysis of a 15‐year‐old patient with typical symptoms of LGMD; they found that *POPDC3* mutations was associated with LGMDR26. In the same year, the results of Zhang et al. [13] demonstrated that a novel splice site variant in POPDC3 resulted in autosomal recessive LGMDR26. In a recent study, de Ridder et al. [14] found that muscle biopsies from patients carrying the *POPDC3* splice site variant showed nonspecific myopathic features, with the presence of a large number of severely atrophied muscle fibres. *POPDC3* variants/knockdown result in LGMDR26, but the specific mechanism remains unclear.

In the study, we found that zebrafish skeletal muscle loss and reduced swimming ability were associated with reduced Popdc3 protein. By using a loss‐of‐function strategy in zebrafish, we demonstrated that loss of *popdc3* deleteriously affects zebrafish mitochondrial function, skeletal muscle mass and swimming ability. Loss of *popdc3* resulted in skeletal muscle mitochondrial dysfunction, which triggered the activation of protein ubiquitination and autophagy autophagy‐lysosomal pathways, leading to decreased muscle mass and swimming ability. This is important to reveal the possible mechanism by which *POPDC3* variants/knockdown causes skeletal muscle atrophy.

## 2 Materials and Methods

### 2.1 Animal Experiments

Wild‐type (WT) male and female zebrafish (AB strain) were purchased from the National Zebrafish Resource Centre (Wuhan, China). *popdc3* knockout (*popdc3* mKO) male and female zebrafish were provided by the research group of Professor Xiushan Wu from the College of Life Sciences of Hunan Normal University. After hybridization between male and female zebrafish, the knockdown effect was verified in the offspring, and 8‐month‐old male zebrafish were used for the experiments. All zebrafish were reared in a specific zebrafish rearing system at the College of Physical Education, Hunan Normal University. The rearing environment was as follows: water temperature 28 ± 1°C, light/dark cycle 14/10 h. Animal experiments followed the Guidelines for Appropriate Behaviour in Animal Experiments (Chinese Scientific Committee) and were approved by the Ethics Committee of Hunan Normal University (Approval No. 2018/046).

### 2.2 Sanger Sequencing

Zebrafish were anaesthetized with MS‐222 (40 mg/L), part of the tail was clipped and DNA was extracted using a DNA extraction kit. The DNA templates are quantified and analysed electrophoretically to ensure concentration, integrity and purity. The extracted DNA was used to configure the PCR system and amplify the *popdc3* gene according to the PCR reaction system. The amplified solution and upstream and downstream primers of the *popdc3* gene were sent to Sangon Biotech for genome sequencing.

### 2.3 Behavioural Tests

Behavioural analysis of zebrafish was carried out using LoliTrack (Denmark). The device is equipped with a USB industrial colour camera that monitors the zebrafish behaviour using the sharp colour contrast between the zebrafish and the surrounding background. Measured indicators include speed, acceleration, travel distance, travel direction, valid/invalid time and exhaustive swimming time. Briefly, zebrafish were placed in glassware containing systematic water and photographed under a microscope after adaptation, a process that was kept quiet and undisturbed. After recording, the videos were imported into the Lolitrack software to analyse the trajectory maps, average velocity and total distance.

### 2.4 Oxygen Consumption (MO_2_) and Critical Swimming Speed (U_crit_)

The MO_2_ and U_crit_ of zebrafish were determined by a Loligo Swimming Respirometer (Denmark), a system equipped with a DAQ‐BT control unit and AutoRespTM software, consisting of a 20‐L tank containing a 170‐mL closed swimming channel for supplying oxygen‐enriched circulating water at 28 ± 0.5°C. Specific methods were according to our previous report [15]. Zebrafish were fasted for 24 h and then placed in the Loligo Swimming Respirometer, acclimatized for 30 min, and then tested according to the appropriate procedures and parameters until zebrafish exhaustion. The measured data were exported, calculated, plotted and analysed.

### 2.5 Histological Analysis

Zebrafish skeletal muscle tissue was cut transversely into thin slices after a series of processes. Transmission electron microscopy (TEM) was used to observe skeletal muscle structure. H&E and wheat germ agglutinin (WGA) staining was performed to observe and calculate skeletal muscle fibre size and cross‐sectional area (CSA). Masson staining was performed to assess collagen deposition. ImageJ software (Version 5.0) was used to calculate the CSA of muscle fibres. The calculation method is as follows: The WGA‐stained muscle fibre image was imported into ImageJ, and the image scale was set according to the scale information in the image. The image is preprocessed, the threshold value is adjusted to distinguish the myofiber from the background and, then, the CSA of the myofiber is automatically measured and labelled using the ‘Analyse Particles’ function with appropriate parameters, and the results are exported and analysed. The mean myofiber CSA was measured in three randomly selected images per zebrafish and calculated at 50–80 fibres per image.

### 2.6 Transcriptome Sequencing and Analysis

The transcriptome sequencing and analysis were conducted by Majorbio Technology Co., Ltd (Shanghai, China). Total skeletal muscle RNA was extracted according to the manufacturer's instructions, and the quality, quantified, of RNA was determined. RNA purification, reverse transcription, library construction and sequencing were performed according to the manufacturer's instructions (Illumina, San Diego, CA). Paired sequencing was performed using an Illumina NovaSeq 6000 platform (Illumina, San Diego, CA, United States). Differential expression analysis was performed using the DESeq2 [16] or DEGseq [17]. DEGs with |log2FC| ≧ 1 and FDR ≤ 0.05 (DESeq2) were considered to be significantly different expressed genes. GO and KEGG functional enrichment analyses were used to determine which DEGs were significantly enriched in GO terms and metabolic pathways compared to a transcriptome‐wide background. GO functional enrichment and KEGG pathway analyses were performed by Goatools and KOBAS [18], respectively.

### 2.7 Determination of Mitochondrial Respiratory

The mitochondrial respiratory parameters of zebrafish skeletal muscle were measured using the Oxygraph‐2 K high‐resolution respirometry system. The muscle tissue was cryogenically ground and added to the sample compartment, and pyruvate, malate, glutamate and ADP were added sequentially to observe the levels of mitochondrial respiratory complex I (Cox I). Cytochrome C was added to assess mitochondrial membrane integrity. Succinate was added to observe the levels of mitochondrial complexes I and II (mitochondrial respiratory complex I + II, Cox I + II). Add the uncoupling agent CCCP, and observe the mitochondrial electron transport chain capacity. Injections of rotenone and antimycin A inhibited Cox I and Cox III.

### 2.8 Transmission Electron Microscopy (TEM)

Zebrafish skeletal muscle tissues were fixed with electron microscope fixative for 24 h. The tissues were washed with saline and fixed in 1% osmium tetroxide solution for 2 h. The tissues were washed, dehydrated and embedded in epoxy resin for fixation. The embedded tissues were polymerized in 65°C oven for 48 h and then cut into 80‐nm slices, stained with uranyl acetate and lead citrate and photographed by transmission electron microscopy. Mitochondria with more than half of their area in the image were considered as one, whereas mitochondria with less than half of their area included were excluded. ImageJ software (Version 5.0) was used to calculate the area and the length of mitochondria. The calculation method is as follows: The representative image of mitochondria was opened in ImageJ, and the image scale was set using the straight‐line tool. Then, use the straight‐line tool to measure the length of the mitochondria or select the appropriate tool to frame the mitochondria measurement area according to the shape of the mitochondria. Finally, the measured data were exported for analysis.

### 2.9 ATP Assay

According to the manufacturer's instructions, the ATP levels in zebrafish skeletal muscle were measured using the ATP assay kit (Cat#A095‐1‐1, Nanjing Institute of Construction Bioengineering, Nanjing, China). Briefly, the tissues were weighed and ground by adding nine times the volume of boiling distilled water to make a 10% tissue homogenate. The homogenate was then boiled for 10 min and centrifuged at 3500 rpm for 10 min, and the ATP assay kit instructions were followed.

### 2.10 Enzyme Assay

Zebrafish skeletal muscle tissues were weighed and ground to make a 10% tissue homogenate. Then, creatine kinase (CK) activity (Cat#A032‐1‐1, Nanjing Institute of Construction Bioengineering, Nanjing, China), lactate dehydrogenase (LDH) activity (Cat#A020‐2‐2, Nanjing Institute of Construction Bioengineering, Nanjing, China), citrate synthase (CS) activity (Cat#A108‐1‐2, Nanjing Institute of Construction Bioengineering, China) and succinate dehydrogenase (SDH) activity (Cat#BC0950, Solarbio, China) were assayed according to the manufacturer's instructions.

### 2.11 ROS Content

The determination of ROS content was detected by DHE staining [15]. DHE enters cells freely and dehydrates in the presence of intracellular superoxide anions to form ethidium bromide. Ethidium bromide binds to RNA or DNA to produce red fluorescence. When the level of superoxide anions in the cell is high, more ethidium bromide is produced, and the red fluorescence is stronger. Briefly, sections were labelled with an immunohistochemistry pen, diluted ROS staining (DHE) solution (D7008, Sigma) was added dropwise to the labelled areas and the cell nuclei were stained with DAPI staining solution. The positive areas were scored by ImageJ software (Version 5.0). The measurements of positive areas are as follows: First, images were converted to greyscale. Thresholds were then set to distinguish positively stained areas from the background, based on visual inspection and comparison with the original images. The selected regions of interest (ROIs) corresponding to DHE‐stained positive areas were automatically delineated. Measurement parameters included area, mean grey value, integrated density, area fraction and limit to threshold, which provided insights into ROS distribution and intensity. The data were obtained in five randomly selected images per zebrafish.

### 2.12 Real‐Time PCR

Total RNA was extracted from the zebrafish skeletal muscle using TRIzol (Thermo Fisher Scientific, United States), according to the manufacturer's protocol. Briefly, the tissues were ground after adding Trizol. Chloroform was added and centrifuged with shaking. The supernatant was taken, and an equal volume of isopropanol was added and centrifuged. The RNA precipitation was washed two times with 75% ethanol, and after the RNA was dry, an appropriate amount of DEPC water was added to measure the concentration and purity of total RNA. Then, the RNA samples were reverse transcribed using the reverse transcription system kit (Takara, Tokyo, Japan). Real‐time PCR was performed using the Bio‐Rad real‐time PCR system (CFX96, Bio‐Rad, Hercules, CA, United States) with a reagent kit from Takara Bio. Reference gene using the gapdh and the relative mRNA expression was determined using the 2^−∆∆CT^ method. The primer sequences used are listed in Table S1.

### 2.13 Western Blot

The total protein extraction of zebrafish skeletal muscle was performed as described previously [15]. Tissues were weighed and added to protein lysate for grinding and lysed for 30 min at low temperatures. After centrifugation, the supernatant was taken, and an equal amount of 1X SDS was added and boiled in a metal bath for 10 min. The protein concentration was measured using the BCA protein assay kit. Proteins were isolated using SDS/PAGE and transferred to a PVDF membrane. The PVDF membrane was closed with 5% milk or BSA for 1 h at room temperature and incubated in primary antibody overnight at 4°C. The next day, after washing the membrane three times with TBST solution, the membrane was incubated for 1 h with the appropriate amount of secondary antibody. After washing the membrane three times, the protein content was detected in a gel imaging analyser. Blots were normalized to GAPDH. ImageJ (NIH, Bethesda, MD, United States) software was used for protein banding analysis. Antibody information is provided in Table S2.

### 2.14 Statistical Analysis

Unpaired *t* tests were used to compare and analyse the data. SPSS 22.0 or GraphPad Prism 7.0 was used to statistically analyse experimental data. Data are mean ± standard deviation (*x* ± *s*), and *p* < 0.05 is considered a statistically significant difference.

## 3 Results

### 3.1 *popdc3* Deficiency Impairs Zebrafish Swimming Ability

**FIGURE 1:**
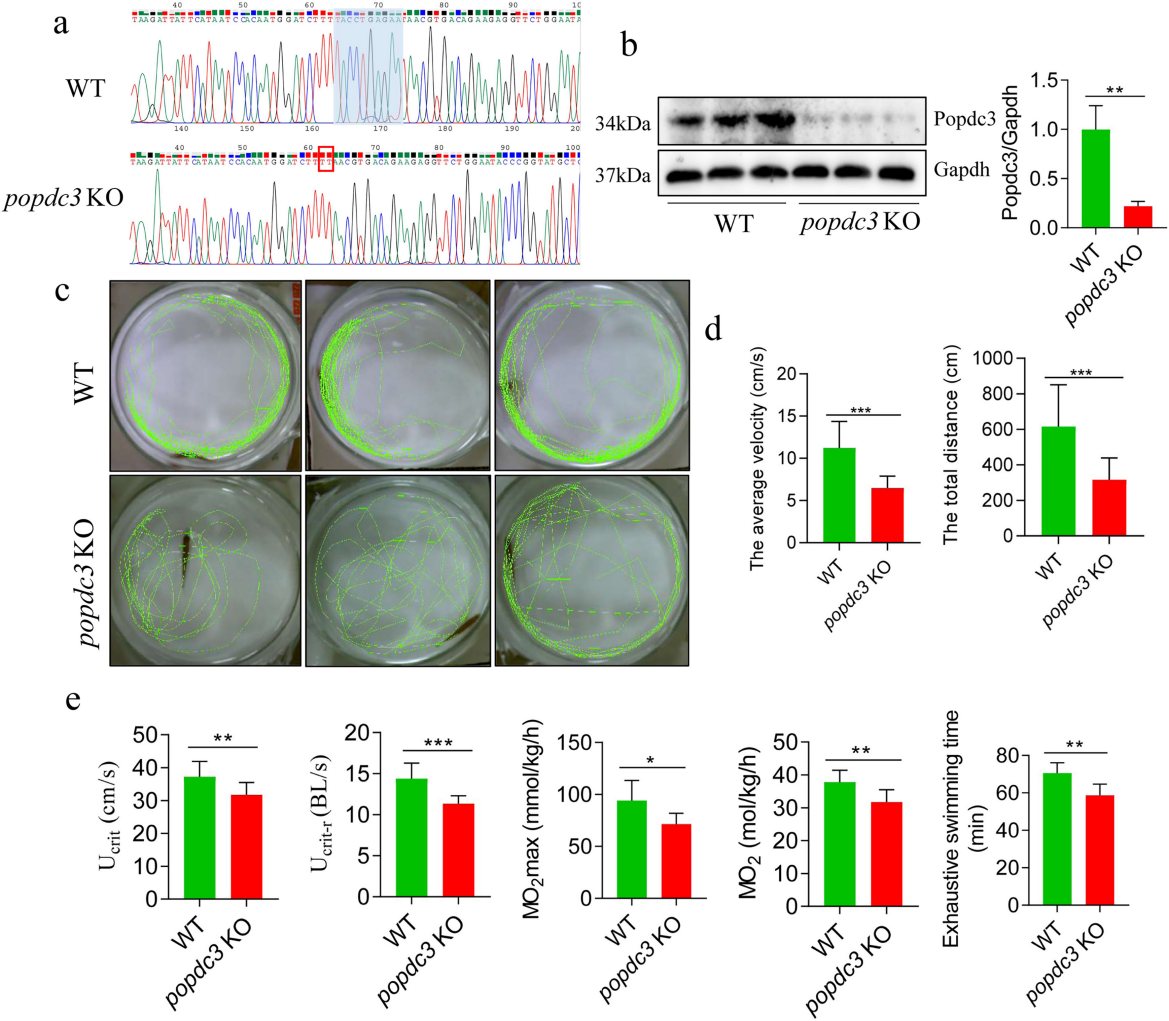
The swimming ability of *popdc3* mKO zebrafish was reduced. (a) Sanger sequencing to verify the *popdc3* knockdown effect (*n* = 6). (b) Western Blotting for Popdc3 protein expression (*n* = 6). (c) Movement trajectories. (d) Average velocity of movement and total distance of movement. (e) U_crit_, U_crit‐r_, MO_2max_, MO_2_ and exhaustive swimming time were determined by the Loligo System (*n* = 15). Data are shown as mean ± SD. *p < 0.05, **p < 0.01 and ***p < 0.001.

First, we verified the *popdc3* knockdown effect by Sanger sequencing. The results showed that compared with the WT zebrafish, *popdc3* mKO zebrafish was deleting 10 bases (TACCTGAGAA) (Figure 1a). According to previous studies, POPDC3 is mainly expressed in skeletal muscle [7, 8]. We detected the Popdc3 protein expression in zebrafish skeletal muscle, and the results showed that zebrafish *popdc3* was successfully knocked down (Figure 1b). Next, we observed the effect of loss of *popdc3* on zebrafish swimming ability. The results revealed that *popdc3* mKO zebrafish behaved poorly, as evidenced by the reluctance to move, fewer movement trajectories (Figure 1c) and significant decreases in average velocity and total distance of movement (Figure 1d). In addition, the U_crit_, relative swimming speed (U_crit‐r_), maximum oxygen consumption (MO_2max_), MO_2_ and exhaustive swimming time during the test phase were significantly reduced in *popdc3* mKO zebrafish (Figure 1e). These indicators are commonly used to indirectly reflect the swimming ability of fish and their swimming performance under increased swimming speed. In conclusion, the results suggest that *popdc3* deficiency results in poor behavioural performance and impairs the swimming ability of zebrafish.

### 3.2 *popdc3* Deficiency Leads to Muscle Loss and Induces Skeletal Muscle Atrophy in Zebrafish

**FIGURE 2:**
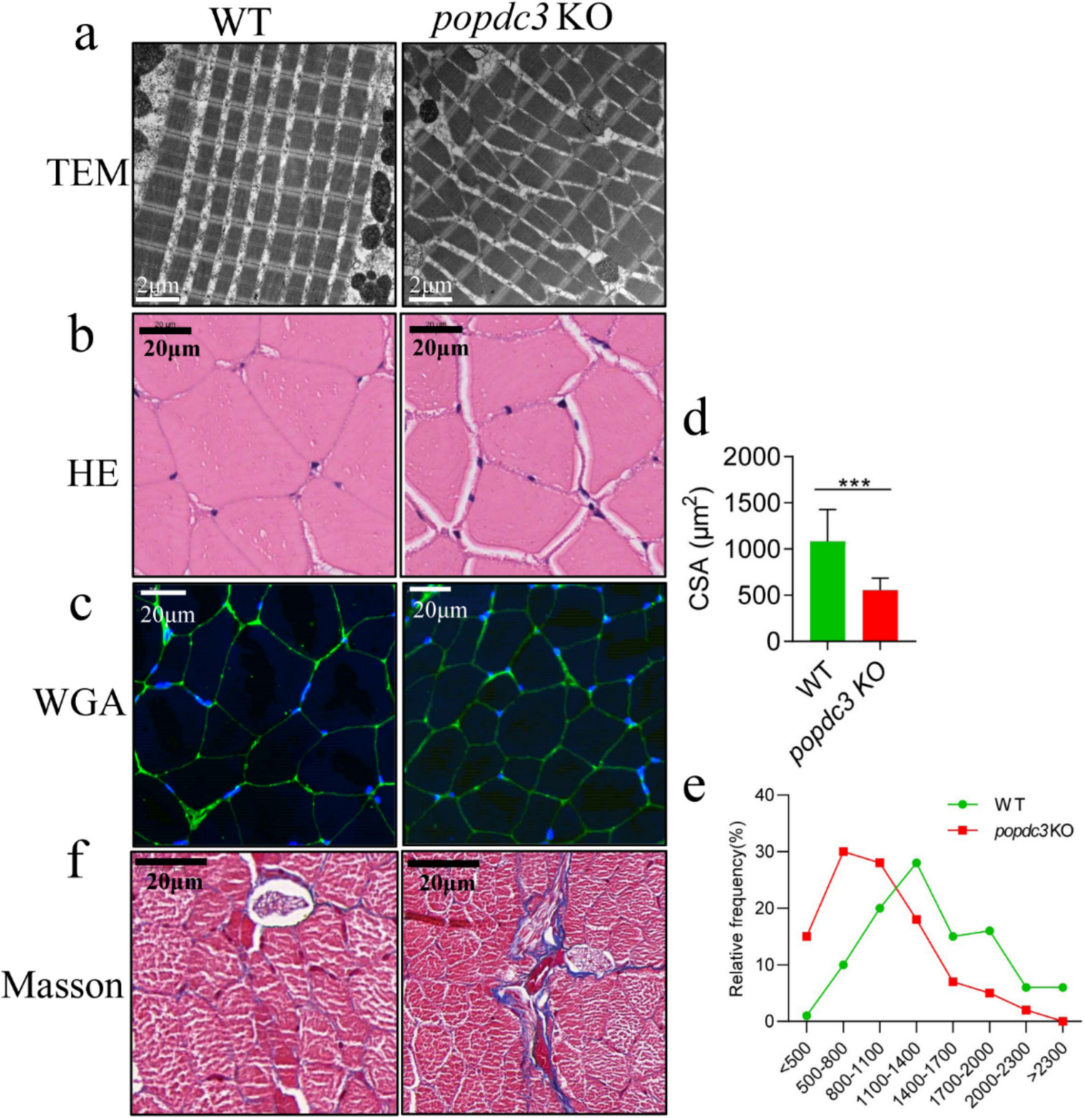
Loss of *popdc3* leads to muscle loss and induces skeletal zebrafish muscle atrophy. (a) TEM to observe the ultrastructure of skeletal muscle, scale bar = 2 μm. (b) Representative micrographs of H&E stained, scale bar = 20 μm. (c) Representative micrographs of WGA stained, scale bar = 20 μm. (d) CSA (based on WGA staining). (e) Frequency distribution of muscle fibre area (%). (f) Masson staining to observe collagen deposition, scale bar = 20 μm. (*n* = 3). Data are shown as mean ± SD. *p < 0.05, **p < 0.01 and ***p < 0.001.

Subsequently, we observed the effects of *popdc3* deficiency on zebrafish skeletal muscle structure and mass. TEM results showed that *popdc3* mKO zebrafish skeletal muscle fibres were disarranged with irregular intermyofibrillar space, light and dark bands of myofibril were not apparent, M‐line degradation, and Z‐line fracture (Figure 2a), the HE and WGA results showed that the CSA and myofiber size were reduced (Figure 2b–d) and the Masson staining results showed that the collagen deposition was increased in *popdc3* mKO zebrafish skeletal muscle (Figure 2f). Statistically, it was found that WT zebrafish myofibril proportion was highest in the range of 1100–1400 um^2^, whereas *popdc3* mKO zebrafish myofibril proportion was highest distributed in the range of 500–800 um^2^ (Figure 2e). In addition, we also found that compared with WT zebrafish, the muscle weight and muscle/body weight of *popdc3* mKO zebrafish were significantly decreased (Figure S1a,b), and the activities of CK and LDH were significantly increased (Figure S1c,d). Collectively, these results suggest that *popdc3* deficiency leads to zebrafish muscle loss and induces skeletal muscle atrophy.

### 3.3 *popdc3* Deficiency Promotes Protein Degradation and Inhibits Protein Synthesis

**FIGURE 3:**
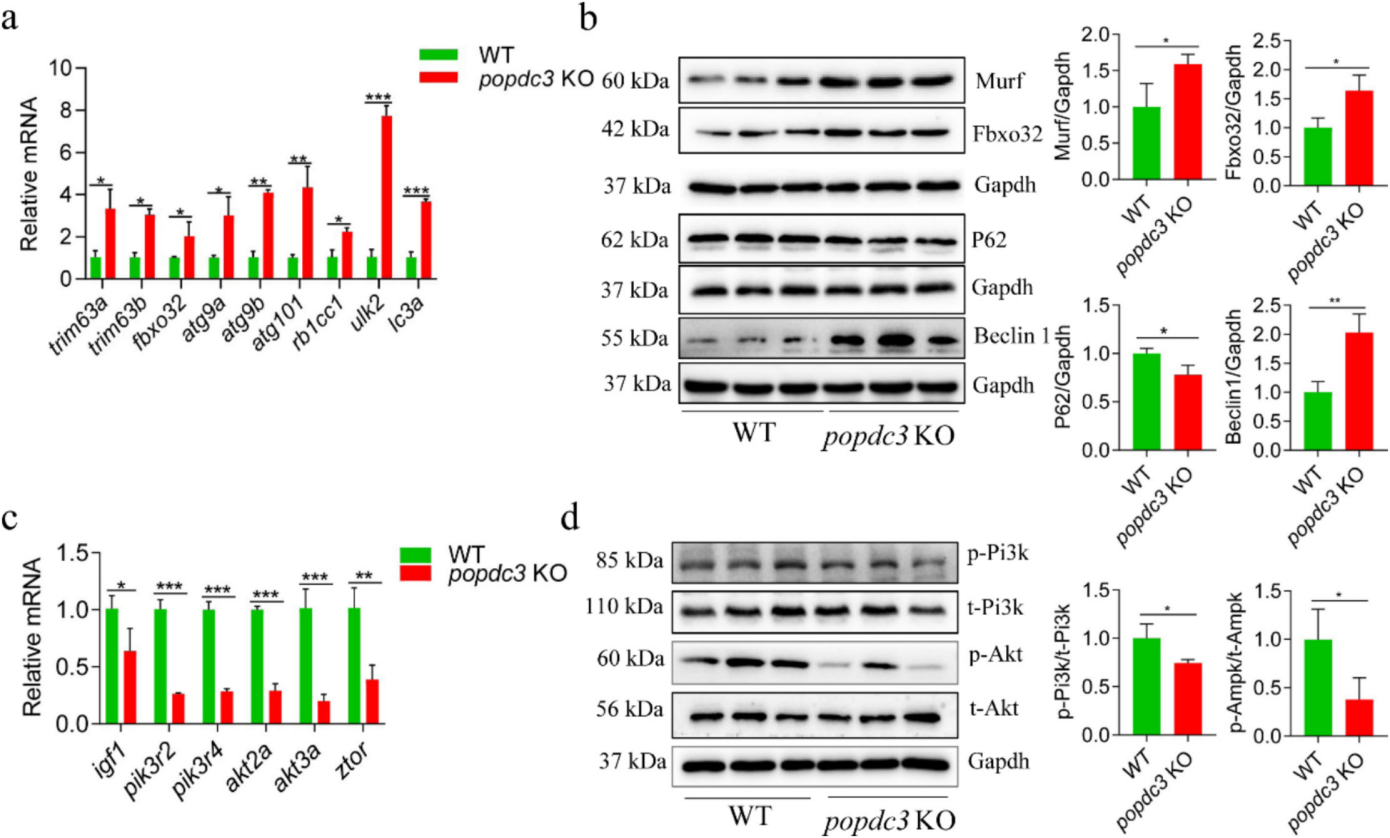
*popdc3* deficiency promotes zebrafish skeletal muscle protein degradation and inhibits protein synthesis. (a) qRT‐PCR to detect the gene expression of *trim63a*, *trim63b*, *fbxo32*, *atg9a*, *atg9b*, *atg101*, *rb1cc1*, *ulk2* and *lc3a*. (b) Western blotting was used to detect the protein expression of Murf and Fbxo2 and autophagy‐related proteins (Beclin1 and P62) expression. (c) qRT‐PCR to detect the gene expression of *igf1*, *pik3r2*, *pik3r4*, *akt2a*, *akt3a* and *ztor*. (d) Western blotting was used to detect the protein expression of p‐Pi3k and p‐Akt (*n* = 6). Data are shown as mean ± SD. *p < 0.05, **p < 0.01 and ***p < 0.001.

Skeletal muscle quality control depends on the dynamic balance between protein synthesis and degradation. To investigate the mechanisms of *popdc3* mKO leading to skeletal muscle atrophy, we analysed the mRNA and protein expression associated with protein degradation (ubiquitination and autophagy). In *popdc3* mKO zebrafish skeletal muscle, we observed increased mRNA abundance and protein expression of Murf (*trim63a* and *trim63b*) and Atrogin‐1 (*fbxo32*), markers of the ubiquitin‐proteasome system (UPS) (Figure 3a,b). The expressions of the autophagic lysosomal pathway (ALP) associated factors were increased, such as the mRNA of *atg9a*, *atg9b*, *atg101*, *rb1cc1*, *ulk2* and *lc3a* and the protein of Beclin1, and autophagy substrate P62 was decreased (Figure 3a,b). Moreover, the expression of IGF‐1/PI3K/AKT, a key signal for protein synthesis was decreased in *popdc3* mKO zebrafish, such as the mRNA of *igf1*, *pik3r2*, *pik3r4*, *akt2a*, *akt3a*, *ztor* and the protein of p‐Pi3k and p‐Akt (Figure 3c–d). The results suggest that loss of *popdc3* promotes zebrafish skeletal muscle protein degradation and inhibits protein synthesis.

### 3.4 RNA‐Seq Analysis of the Mechanism of *popdc3* Deficiency‐Induced Zebrafish Muscle Atrophy

**FIGURE 4:**
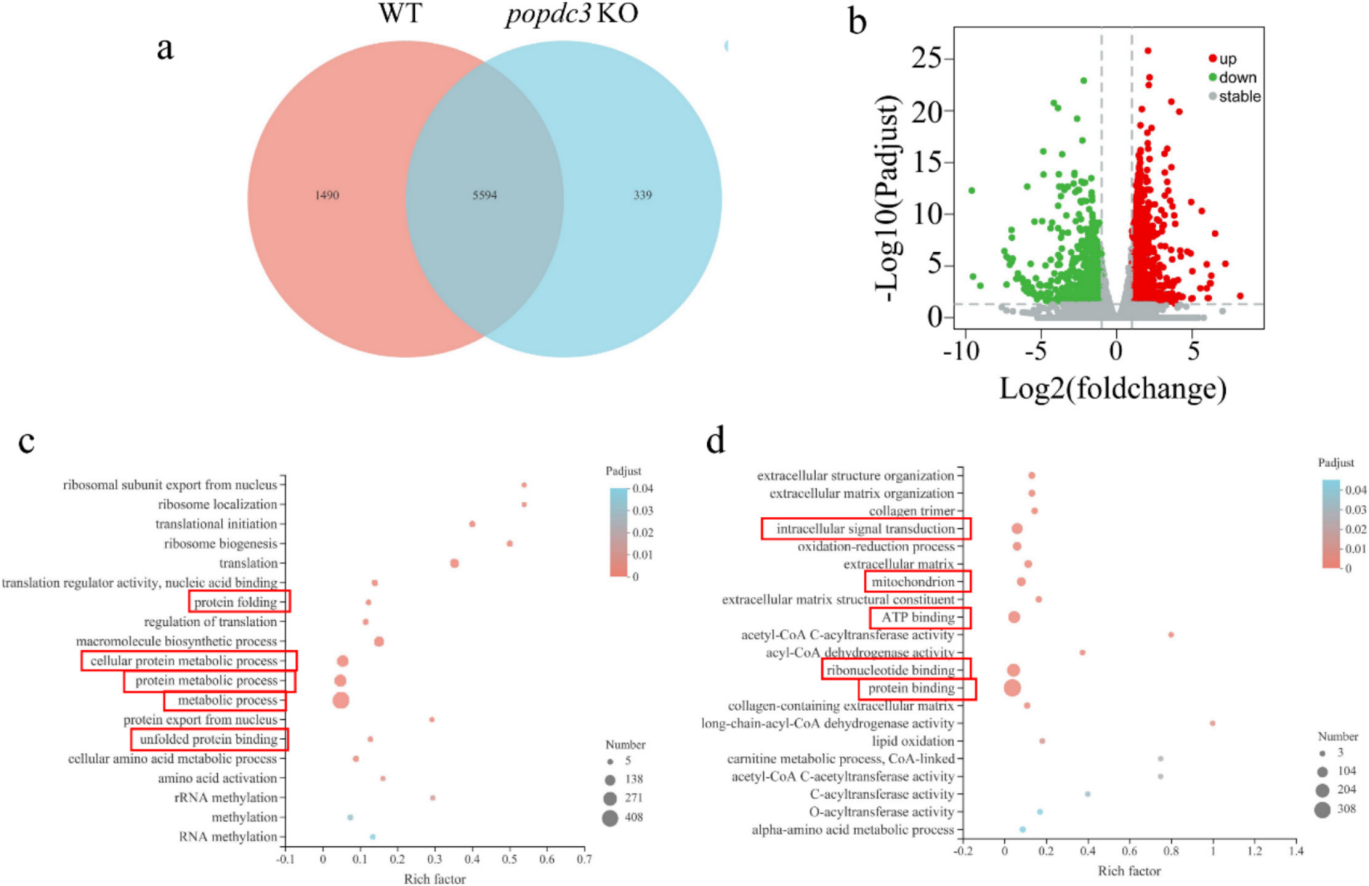
RNA‐Seq analysis of the mechanism of muscle atrophy in *popdc3* mKO zebrafish. (a) Venn diagram of the total number of identified genes. (b) Volcano plot constructed using fold change values and Padi value. Red: upregulated; green: downregulated; filtered: nonsignificant. (c) GO terms enriched in upregulated genes. (d) GO terms enriched in downregulated genes (*n* = 3).

To fully understand the altered gene expression profiles and the mechanisms of *popdc3* mKO zebrafish skeletal muscle, we analysed the skeletal muscle transcriptomes of WT and *popdc3* mKO zebrafish by RNA sequencing. The results showed that 339 genes were specifically expressed and 1490 genes were not detected in the *popdc3* mKO zebrafish skeletal muscle compared to WT zebrafish (Figure 4a). A total of 1820 differentially expressed genes existed between the two groups (Padj < 0.05, fold change > 2). Among them, 952 genes were upregulated, and 868 genes were downregulated (Figures 4b and S2a,b). GO enrichment analysis showed that the enriched terms of upregulated genes were mainly concentrated in metabolic processes, protein metabolism processes, cellular protein metabolism processes, protein folding and unfolded protein binding (Figure 4c). Downregulated genes were enriched in intracellular signal transduction, ribonucleotide binding, ATP‐binding, mitochondrial and protein‐binding (Figure 4d). GO and KEGG enrichment analyses of differentially expressed genes revealed that mitochondria‐related genes and pathways were significantly repressed in *popdc3* mKO zebrafish skeletal muscle. GSEA analyses showed that mitochondrial transmembrane transport, mitochondrion organization, mitochondrial membrane, mitochondrial membrane part, mitochondrial inner membrane, oxidative phosphorylation, glycolysis/gluconeogenesis, propanoate metabolism, citrate cycle (TCA cycle) and pyruvate metabolism were significantly inhibited in *popdc3* mKO zebrafish skeletal muscle (Figure S3a,b and Table S3). These suggest that *popdc3* mKO zebrafish skeletal muscle mitochondrial function is decreased or impaired.

### 3.5 *popdc3* Deficiency Leads to Severe Mitochondrial Structural and Respiratory Function Abnormalities in Zebrafish Skeletal Muscle

**FIGURE 5:**
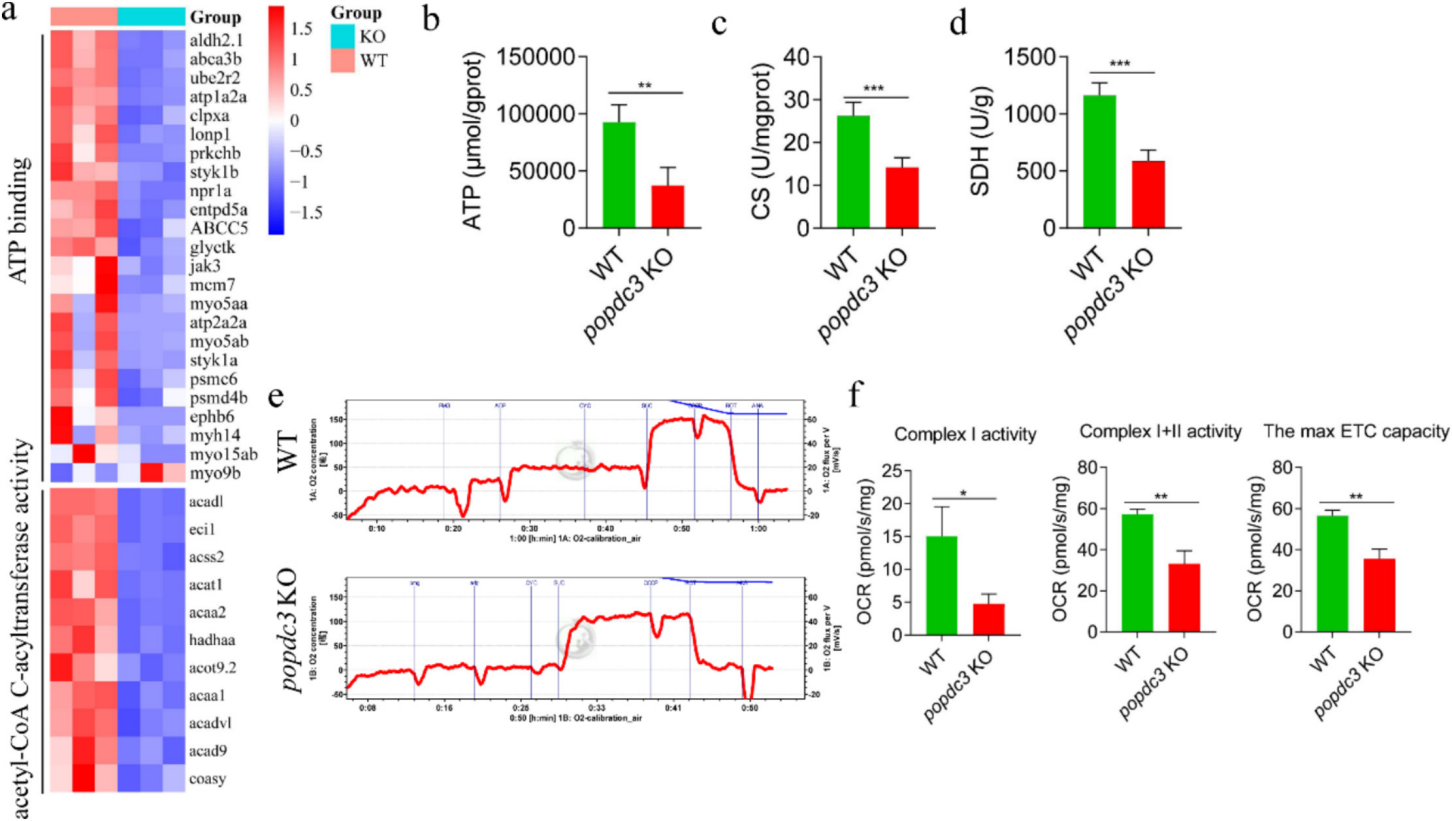
*popdc3* deficiency reduces mitochondrial respiratory function in zebrafish skeletal muscle. (a) Heatmap of differentially expressed genes (*n* = 3). (b) ATP content. (c, d) CS and SDH activities. (e) Real‐time oxygen consumption rate profile of mitochondrial respiration. (f) Activity of Cox I. (g) Activity of Cox I + II. (h) The max ETC capacity (*n* = 10). Data are shown as mean ± SD. *p < 0.05, **p < 0.01 and ***p < 0.001.

RNA‐Seq showed that genes involved in the regulation of ATP binding and acetyl‐CoA C‐acyltransferase activity were significantly downregulated in *popdc3* mKO zebrafish (Figure 5a). Further, we found that ATP content, citrate synthase (CS) activities and succinate dehydrogenase (SDH) activities were significantly decreased in *popdc3* mKO zebrafish skeletal muscle (Figure 5b–d). The activities of mitochondrial complex I (Cox I) and mitochondrial complex I + II (Cox I + II) and maximal electron transport chain (ECT) capacity were significantly reduced (Figure 5e,f). In conclusion, the results suggest that *popdc3* deficiency leads to abnormal mitochondrial respiratory function in zebrafish skeletal muscle.

### 3.6 *popdc3* Deficiency Impairs Mitochondrial Structure and Biogenesis in Zebrafish Skeletal Muscle

**FIGURE 6:**
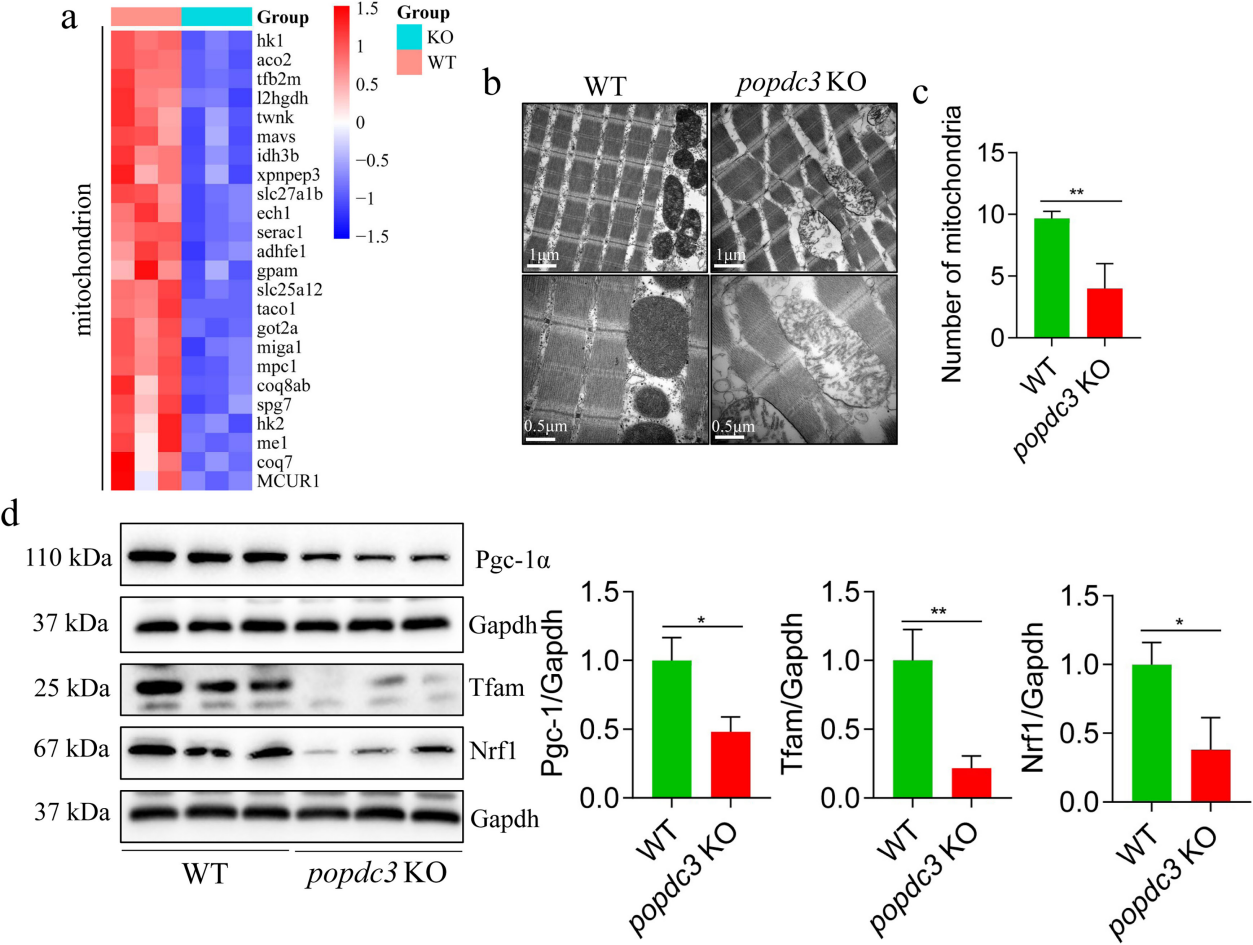
Loss of *popdc3* impairs mitochondrial structure and biogenesis in zebrafish skeletal muscle. (a) Heatmap of differentially expressed genes (*n* = 3). (b) Representative images of mitochondriaTEM, scale bars = 1 and 0.5 μm. (c) Mitochondrial number. (d) Mitochondrial area. (e) Mitochondrial length. (f) Western blotting to detect the protein expressions of Nrf1, Tfam and Pgc‐1α (*n* = 6). Data are shown as mean ± SD. *p < 0.05 and **p < 0.01.

Next, we determined whether *popdc3* mKO impairs mitochondrial structure and biogenesis. The results revealed that genes involved in the regulation of mitochondrial function were downregulated (Figure 6a). TEM showed that the skeletal muscle mitochondria of WT zebrafish were short rod or globular, with a high number of mitochondria, abundant and well‐arranged cristae, and a dense mitochondrial matrix. However, *popdc3* mKO zebrafish skeletal muscle mitochondria were severely abnormal, showing swollen, ruptured membranes, missing cristae, matrix lysis reduced numbers and increased mitochondrial area and length (Figure 6b–e). Western blotting results showed that the protein expressions of the major regulators of mitochondrial biogenesis, Pgc‐1α, Tfam and Nrf1, were significantly decreased in *popdc3* mKO zebrafish (Figure 6f). These results suggest that *popdc3* deficiency impairs mitochondrial structure and reduces mitochondrial biogenesis in zebrafish skeletal muscle.

### 3.7 *popdc3* Deficiency Impairs Mitochondrial Kinetic Homeostasis in Zebrafish Skeletal Muscle

**FIGURE 7:**
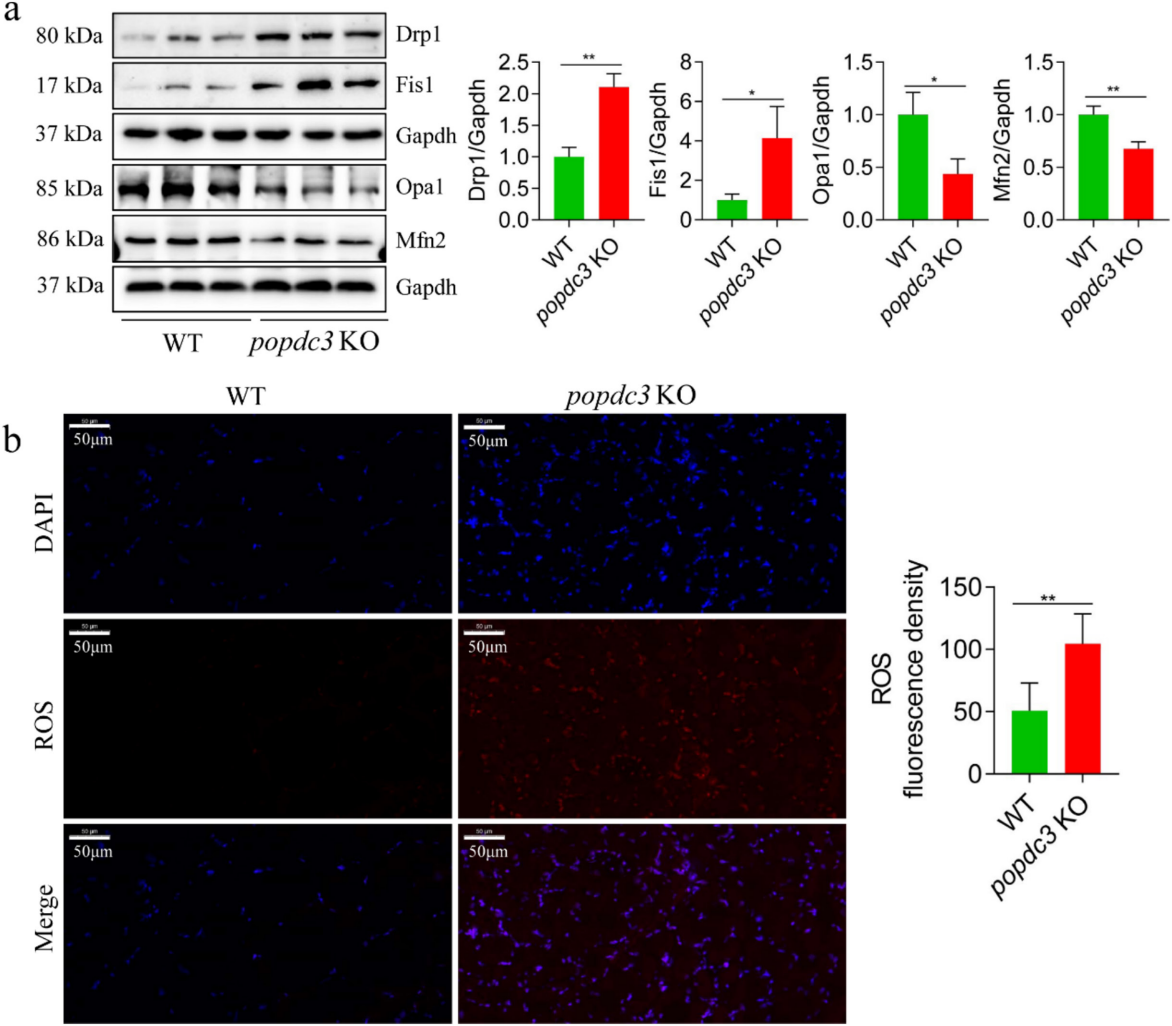
*popdc3* deficiency impairs mitochondrial quality control processes in zebrafish skeletal muscle. (a) Western blotting to detect the protein expressions of Drp1, Fis1, Opa1 and Mfn2 (*n* = 6). (b) DHE staining (*n* = 3). Data are shown as mean ± SD. *p < 0.05 and **p < 0.01.

The results showed that the factors associated with mitochondrial fusion, such as Opa1 and Mfn2, were significantly decreased, whereas Drp1, Mff and Fis1, which are associated with mitochondrial fission, were increased in *popdc3* mKO zebrafish skeletal muscle (Figure 7a). In addition, we examined ROS content and found that ROS levels were significantly higher in *popdc3* mKO zebrafish skeletal muscle than in WT zebrafish (Figure 7b). In conclusion, these results suggest that loss of *popdc3* results in decreased or impaired function of zebrafish skeletal muscle.

## 4 Discussion

**FIGURE 8:**
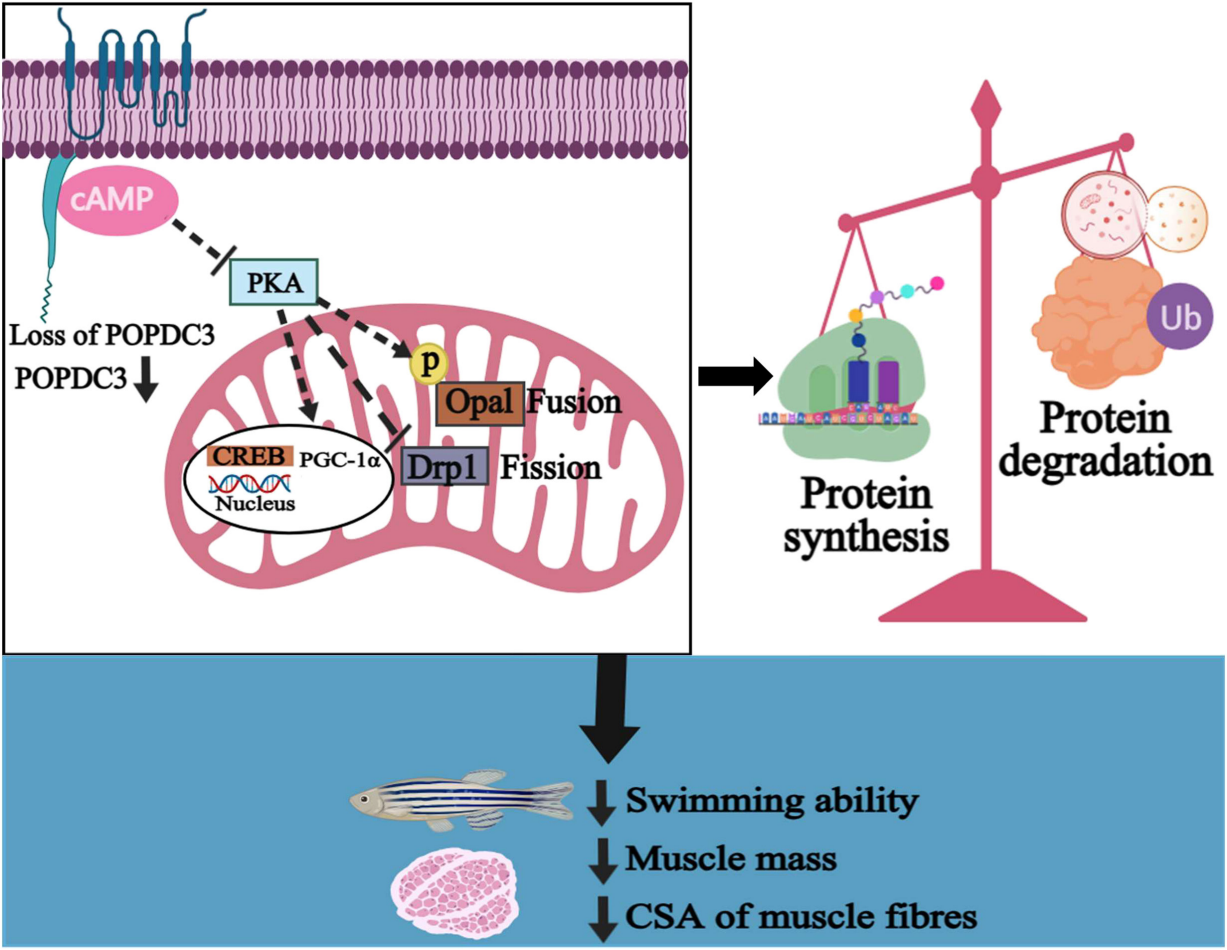
The mechanism of POPDC3 in regulating mitochondrial function and skeletal muscle mass in response to skeletal muscle atrophy.

Although previous studies have shown that *POPDC3* variants/knockout cause LGMDR26, the underlying pathological mechanisms remain unknown. In this study, we describe the importance of POPDC3 in regulating mitochondrial function and skeletal muscle mass in response to skeletal muscle atrophy (Figure 8). We found that POPDC3 is important in maintaining skeletal muscle mass, strength and function. Loss of *popdc3* leads to a significant reduction in skeletal muscle mass and zebrafish swimming ability. Importantly, our data suggest that *popdc3* mKO zebrafish mitochondrial function is impaired, as evidenced by decreased mitochondrial respiration and biogenesis and imbalanced kinetics. This may be one of the factors causing the imbalance between protein synthesis and degradation in skeletal muscle. Thus, these findings reveal an important role for POPDC3 in the protection of mitochondrial function and maintenance of skeletal muscle mass and strength and the pathological mechanism by which *POPDC3* variants/knockout leads to skeletal muscle atrophy.

Human genetic disorders associated with *POPDC3* variants highlight the importance of POPDC3 in the maintenance of skeletal muscle mass and function. *POPDC3* variant/knockout is associated with LGMDR26, a primary skeletal muscle atrophy [13]. Skeletal muscle atrophy is directly caused by an imbalance between protein synthesis and degradation and is mainly characterized by the reduction and atrophy in muscle mass and fibre CSA at the histological level [19]. Patients experience reduced exercise ability, easy fatigue and decreased quality of life [1, 20]. Our previous study found that a variety of factors induce reduced protein synthesis and/or increased degradation, leading to reduced muscle fibre CSA, muscle atrophy and reduced swimming ability in zebrafish, such as ageing [15], alcohol [21], dexamethasone, D‐galactose [22] and obesity [23]. In this research, we found that loss of *popdc3* leads to decreased skeletal muscle mass and downregulation of the IGF‐I/PI3K/AKT/mTOR pathway and increases in the factors' expression related to UPS and ALP. Notably, the behavioural performance and swimming ability of *popdc3* mKO zebrafish were reduced, as evidenced by the reluctance to move, fewer movement trajectories and significant decreases in average velocity, total distance of movement, U_crit_, U_crit‐r_, MO_2_max, MO_2_ and exhaustive swimming time.

In zebrafish, *popdc3* knockdown results in phenotypes such as curled tail and muscle dystrophic [12]. The severe phenotypes in *popdc3* knockdown zebrafish might be due to the complete loss of the protein. In muscle biopsies of patients, it has been observed the mutant proteins accumulate at the perinuclear level, suggesting that pathologic mechanisms could be due to the lack of the protein at the plasma membrane (sarcolemma and T‐tubules) and the mislocalization of the pathogenic variant inside the myoblasts. Therefore, complete loss of the protein may disrupt the normal physiological function of POPDC3 in muscle tissues, leading to severe disease phenotypes. However, more in‐depth studies are needed to determine the extent to which these phenotypes represent the LGMD pathology. LGMD is a group of hereditary muscle disorders characterized by muscle atrophy and hypomuscularity, and the pathological mechanisms are complex, involving the interaction of multiple genes and proteins. Therefore, *popdc3* knockdown may be only one part of the pathological mechanism of LGMDR26; the underlying in vivo physiological relevance and mechanisms remain largely convoluted. The Popeye structural domain of the POPDC protein was found to be an evolutionarily highly conserved structural feature, with secondary structure predictions showing similarity to the PKA cyclic nucleotide structural domain. Additionally, the POPDC proteins exhibit a high affinity for binding cAMP that is comparable to that of protein kinase A (PKA) [11, 24]. The cAMP signalling pathway is involved in the regulation of a variety of physiological and pathological processes. cAMP phosphorylates its major downstream target PKA, and phosphorylated PKA activates the cAMP effector binding protein (CREB), which is involved in the regulation of mitochondrial function [25]. It suggests that POPDC3 regulates mitochondrial function through binding to cAMP.

The primary function of mitochondria depends on the production of ATP from oxidative phosphorylation (OXPHOS) within the ETC, which is essential for lipid and protein metabolism, skeletal muscle contraction and cell survival [26, 27]. Specifically, Cox I‐IV of the mitochondrial respiratory on the mitochondrial inner membrane converts free energy into potential energy by catalysing electron transfer and driving ATP synthase to synthesize ATP [28]. However, a variety of stresses (ageing, hypoxia, starvation, inflammation, prolonged bed rest or lack of physical activity, obesity and cancer cachexia) induced mitochondrial function abnormalities, such as decreased mitochondrial respiratory chain complex activity, mitochondrial swelling, membrane rupture, reduced number and reduced ATP content [29]. In the study, RNA‐Seq analysis revealed that loss of *popdc3* leads to significant downregulation of genes and pathways related to mitochondria, acetyl‐CoA C‐acyltransferase activity and ATP binding in zebrafish skeletal muscle. Second, *popdc3* mKO zebrafish skeletal muscle mitochondria were severely abnormal, as evidenced by mitochondrial swelling, reduced number, membrane rupture, cristae deletion, matrix dissolution, a significant reduction of ATP content, CS and SDH activities and a decrease in mitochondrial Cox I, Cox I + II activities and maximal ETC capacity. This suggests that mitochondrial function is impaired in the *popdc3* mKO zebrafish skeletal muscle. As a dynamic organelle, mitochondria can be appropriately regulated in response to external stresses to maintain their homeostasis, which depends on several key processes, including mitochondrial fission and fusion, autophagy and biogenesis [30]. It is commonly believed that reducing mitochondrial fission or increasing mitochondrial fusion helps to maintain mtDNA stability and prevents the loss of essential mitochondrial components [31]. Disruption of mitochondrial fusion increases mitochondrial dysfunction, leading to muscular dystrophy and neurodegenerative diseases [32, 33]. In the present study, we found that loss of *popdc3* leads to reduced mitochondrial biogenesis, kinetic imbalance (increased fission and decreased fusion) and excessive accumulation of ROS in zebrafish skeletal muscle.

In conclusion, our results suggest that POPDC3 is an important protein responsible for maintaining skeletal muscle mass and mitochondrial function. *popdc3* deficiency leads to severe skeletal muscle mitochondrial dysfunction and decreased CSA and mass of muscle. *popdc3* deficiency results in the decrease of zebrafish swimming ability. However, this study has some limitations: (1) Skeletal muscle‐specific *popdc3* mKO zebrafish were not used in our study. (2) The effect of *popdc3* deficiency on mitochondrial autophagic flux in skeletal muscle was not assessed. (3) In *popdc3* mKO zebrafish skeletal muscle, mitochondrial dysfunction and reduced ATP production suggest impaired muscle metabolism. Whether *popdc3* mKO causes fatty infiltration and altered fibre type ratios needs to be further explored. Despite these limitations, we believe that our study provides new insights into understanding the mechanisms by which *popdc3* deficiency induces LGMDR26.

## Consent

All coauthors consented to the publication of the research.

## Conflicts of Interest

The authors declare no conflicts of interest.

## Supporting information


**Figure S1** (a) Skeletal muscle weight. (b) Muscle weight ratio. (c‐d) The CK and LDH content in skeletal muscle (*n* = 10). Data are shown as mean ± SD. ** *P* < 0.01, *** *P* < 0.001.
**Figure S2** The up‐and down‐regulation of differentially expressed genes (*n*=3).
**Figure S3** GSEA of Mitochondrial function (n=3). (a) mitochondrial transmembrane transport; mitochondrion organization; mitochondrial membrane; mitochondrial membrane part; mitochondrial inner membrane. (b) oxidative phosphorylation; glycolysis/gluconeogenesis; propanoate metabolism; GSEA of citrate cycle (TCA cycle); pyruvate metabolism (*n*=3).

**Table S1** List of primers used for RT‐qPCR.
**Table S2** Antibody sources and dilutions.
**Table S3** A GSEA analyses of GO and KEGG.

## Data Availability

Data used to support the findings of this study are available from the corresponding author upon request.
