# Supplementary material for: Loss of popdc3 Impairs Mitochondrial Function and Causes Skeletal Muscle Atrophy and Reduced Swimming Ability in Zebrafish
Source: J Cachexia Sarcopenia Muscle. 2025 Apr 16;16(2):e13794. doi: 10.1002/jcsm.13794 (PMC12001256; doi:10.1002/jcsm.13794)
Supplement: Supplementary file 1 — Figure S1 (a) Skeletal muscle weight. (b) Muscle weight ratio. (c‐d) The CK and LDH content in skeletal muscle (n = 10). Data are shown as mean ± SD. ** P < 0.01, *** P < 0.001. Figure S2 The up‐and down‐regulation of differentially expressed genes (n=3). Figure S3 GSEA of Mitochondrial function (n=3). (a) mitochondrial transmembrane transport; mitochondrion organization; mitochondrial membrane; mitochondrial membrane part; mitochondrial inner membrane. (b) oxidative phosphorylation; glycolysis/gluconeogenesis; propanoate metabolism; GSEA of citrate cycle (TCA cycle); pyruvate metabolism (n=3). [file JCSM-16-e13794-s002.docx]

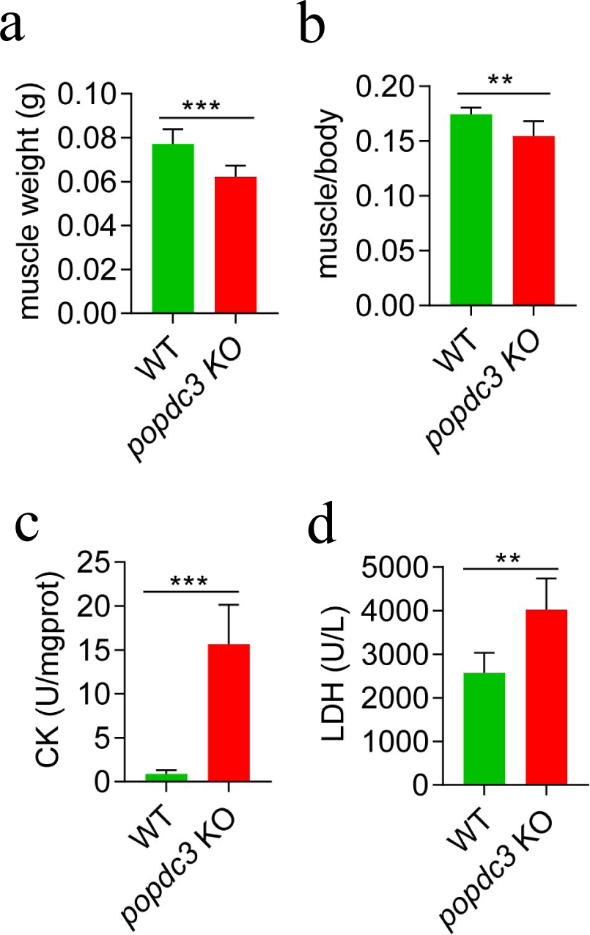


**Supplementary figure 1.** (a) Skeletal muscle weight. (b) Muscle weight ratio. (c-d) The CK and LDH content in skeletal muscle (n = 10). Data are shown as mean ± SD. ** P < 0.01, *** P < 0.001.


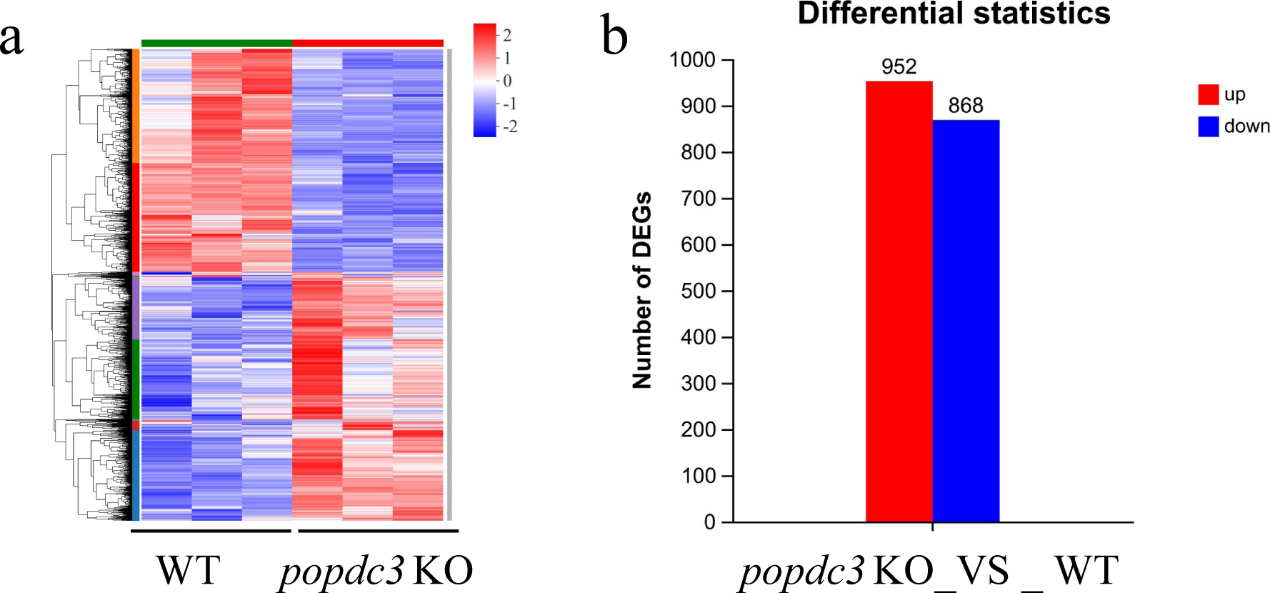


**Supplementary figure2.** The up-and down-regulation of differentially expressed genes (n=3).


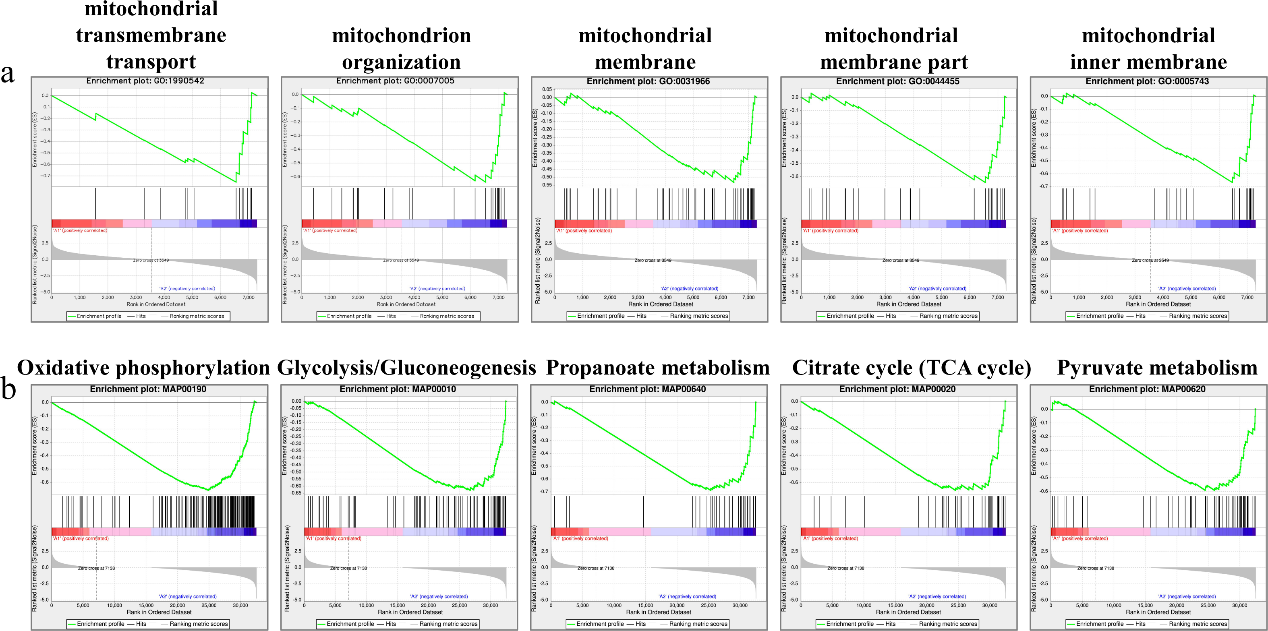


**Supplementary figure 3.** GSEA of Mitochondrial function (n=3). (a) mitochondrial transmembrane transport; mitochondrion organization; mitochondrial membrane; mitochondrial membrane part; mitochondrial inner membrane. (b) oxidative phosphorylation; glycolysis/gluconeogenesis; propanoate metabolism; GSEA of citrate cycle (TCA cycle); pyruvate metabolism (n=3).
