# Supplementary material for: Loss of popdc3 Impairs Mitochondrial Function and Causes Skeletal Muscle Atrophy and Reduced Swimming Ability in Zebrafish
Source: J Cachexia Sarcopenia Muscle. 2025 Apr 16;16(2):e13794. doi: 10.1002/jcsm.13794 (PMC12001256; doi:10.1002/jcsm.13794)
Supplement: Supplementary file 2 — Table S1 List of primers used for RT‐qPCR. Table S2 Antibody sources and dilutions. Table S3 A GSEA analyses of GO and KEGG. [file JCSM-16-e13794-s001.docx]

Supplementary Table 1. List of primers used for RT-qPCR

| Gene | Accession number | Forward primer (5′–3′) | Reverse primer (5′–3′) |
| --- | --- | --- | --- |
| akt2a | NM_198146.2 | CCGCACCAAAGTGACCATGA | GCGTAGGATCTTCATGGCGT |
| akt3a | XM_021480597.1 | TAGATGCCTACAGTGGACTAC | CCATGTTGTCTATCTGTGAGG |
| fbxo32 | NM_200917.1 | CACAGACAGACAGATCCGCAA | GCAGTGCAAGGATGGTCTGT |
| gapdh | NM_001115114.1 | ATCATCTCTGCCCCAAGTGC | ACGGTCTTCTGTGTTGCTGT |
| igf1 | NM_001172627.1 | CATTGCCCGCATCTCATCCT | GACATCACACCAATGCCCCT |
| atg9a  atg9b  atg101  rb1cc1  ulk2  lc3a | NM_001083031.1  NM_001320078.1  NM_001037239.2  XM_009302198.3  XM_002664615.5  NM_000546.6 | ATGACATCCTCTTCGCCAACAA  TTCACTACATGCCCGACCAC  ACGTGGACTGCGACTTCATA  ACACTACAGACAATGGGCGA  TACGCATCAAGATCGCCGAC  CCATCCGACAGACCCTTCAA | ACACTGTCACGGATCCTAGC  CCTCCAAGATGAAGACCGCT  GAATTGCCAAGCGCATCCTTAAA  TCCAGTCCTCGAAACAAGCG  ACAGGTCAGCTTTGGCATCA  ATACCTCTCAATGATCACCGGAA |
| pik3r4 | XM_005158299.4 | AAGAGAGCTCGCAAACATACG | TCCACTGTCTGGAATCGTCG |
| pik3r2 | NM_212822.2 | CAGCCTCCTTGCAGGTTGTT | CTCGGTCGCTGATTGTCAGA |
| trim63a | NM_001002133.1 | ATTCTCCCGTGCCAACACAA | CGAAGCGACAAGTAGGGCAT |
| trim63b | NM_201095.1 | CTTCAGAGGAATCTTCTTGTTG | GAGTAACGCAGTAGATGTTGA |
| ztor | NM_001077211.2 | GCCATTCAGATCATCAACCGAGT | GCGATGCCGATTCCCACTCT |

Supplementary Table 2. Antibody sources and dil utions.

| Antibodies | Source | Catalog | Dilution ratio |
| --- | --- | --- | --- |
| rabbit anti-Popdc3 anti body  rabbit anti-Beclin1 anti body  rabbit anti-Murf anti body  rabbit anti- Fbxo32 anti body  rabbit anti-t-Akt anti body  rabbit anti-p-Akt anti body  rabbit anti-t-Pi3k anti body  rabbit anti-p-Pi3k anti body  rabbit anti-Mfn2 anti body  rabbit anti-Opa1 anti body  rabbit anti-Drp1 anti body  rabbit anti-Fis1 anti body  mouse anti-Pgc-1α anti body  rabbit anti-Nrf1 anti body  rabbit anti-Tfam anti body  rabbit anti-P62 anti body | Proteintech, Wuhan, China  Proteintech, Wuhan, China  Abcam, Boston, USA  Abcam, Boston, USA  Proteintech, Wuhan, China  CST, Boston, USA  Proteintech, Wuhan, China  CST, Boston, USA  Proteintech, Wuhan, China  Proteintech, Wuhan, China  Proteintech, Wuhan, China  Proteintech, Wuhan, China  Proteintech, Wuhan, China  Proteintech, Wuhan, China  Proteintech, Wuhan, China  Proteintech, Wuhan, China | 26276-1-AP  11306-1-AP  ab172479  ab168372  10176-2-AP  #4060  20584-1-AP  #4228  12186-1-AP  27733-1-AP  12957-1-AP  10956-1-AP  66369-1-Ig  12482-1-AP  22586-1-AP  18420-1-AP | 1:2000  1:2000  1:2000  1:2000  1:2000  1:2000  1:2000  1:2000  1:2000  1:2000  1:2000  1:2000  1:2000  1:2000  1:2000  1:1500 |
| rabbit anti-GAPDH antibody | Servicebio, Wuhan, China | GB11002 | 1:2000 |

Supplementary Table 3. A GSEA analyses of GO and KEGG.

| gene set name | Description | Size | ES | NES | p-adjust | Rank at MAX | Leading edge |
| --- | --- | --- | --- | --- | --- | --- | --- |
| GO:1990542 | mitochondrial transmembrane transport | 15 | -0.75 | -1.91 | 0.015 | 741 | 8 |
| GO:0007005 | mitochondrion organization | 25 | -0.64 | -1.76 | 0.041 | 764 | 11 |
| GO:0044455 | mitochondrial membrane part | 27 | -0.64 | -1.84 | 0.008 | 741 | 12 |
| GO:0031966 | mitochondrial membrane | 51 | -0.533 | -1.734 | 0.020 | 828 | 17 |
| GO:0005743 | mitochondrial inner membrane | 27 | -0.668 | -1.891 | 0.003 | 828 | 13 |
| MAP00190 | Oxidative phosphorylation | 153 | -0.660 | -1.608 | 0.002 | 7627 | 98 |
| MAP00640 | Propanoate metabolism | 35 | -0.688 | -1.527 | 0.01563 | 7165 | 27 |
| MAP00010 | Glycolysis/Gluconeogenesis | 87 | -0.630 | -1.490 | 0.027 | 5801 | 41 |
| MAP00020 | Citrate cycle (TCA cycle) | 37 | -0.657 | -1.451 | 0.037 | 7973 | 25 |
| MAP00620 | Pyruvate metabolism | 54 | -0.592 | -1.365 | 0.015 | 7165 | 35 |
